# Factors Associated With Patient Portal Engagement in Otolaryngology

**DOI:** 10.1002/oto2.70050

**Published:** 2024-11-26

**Authors:** Jesse K. Siegel, Chloe Verducci, Agnes Hurtuk

**Affiliations:** ^1^ Department of Otolaryngology–Head and Neck Surgery Loyola University Medical Center Maywood Illinois USA; ^2^ Department of Otolaryngology–Head and Neck Surgery University of Massachusetts Medical Center Worcester Massachusetts USA

**Keywords:** business of medicine, health equity, patient engagement, patient experience, patient portal

## Abstract

**Objective:**

Online patient portals are important tools for patient engagement, and their use has increased particularly since the COVID‐19 pandemic and the 21st Century Cures Act. However, prior work across various specialties has demonstrated disparities in patient portal usage with respect to age, gender, race, and insurance status. However, this has not been studied in the field of otolaryngology, and not since the onset of the COVID‐19 pandemic.

**Study Design:**

Retrospective study.

**Setting:**

Single tertiary care outpatient otolaryngology practice over a 3‐year period (December 2019‐December 2022).

**Methods:**

We used univariate and multivariate analyses to measure how patient portal use for appointment scheduling varied before and after the COVID‐19 pandemic and across demographic groups including gender, race, age, primary language, insurance type, and primary care physician (PCP) status (Loyola vs non‐Loyola).

**Results:**

From December 2019 to December 2022, 49,462 unique patients scheduled 221,611 otolaryngology clinic visits. Significantly more online scheduling occurred after the onset of the COVID‐19 pandemic (10.7% vs 1.9%, *P* < .01). In multivariate analysis, male gender, age <18 or >65 years, non‐English primary language, and outside PCP were all associated with a lower likelihood of online appointment scheduling (*P* < .01 for each). Patients with Medicare had higher odds of portal use than commercially insured patients when controlling for other demographic variables (*P* = .034).

**Conclusion:**

Patient portal use in otolaryngology has markedly increased over the last 3 years, but utilization varies across demographic groups. This provides an opportunity to improve patient portal content and outreach, and ultimately make portals more accessible to diverse patient populations.

Establishing timely, affordable, and equitable access to health care has been an increasingly prominent effort over the last several decades, gaining further momentum with the emergence of the COVID‐19 pandemic and many of the changes within the health care industry. One innovation for improving patient access to care has been online patient portals, on which patients can schedule appointments, view results, and communicate remotely with health care providers.

The onset of the COVID‐19 pandemic rapidly and dramatically increased interest in and use of online portals.^1^, ^2^ Additionally, in 2021, a US federal rule was signed into law requiring all US health care providers to give patients access to all health information in their electronic medical records without charge. Known as the “information sharing rule,” this mandates that patients have rapid, free, and full access upon request to test results, medication lists, referral information, and clinical notes, all in electronic formats.^3^

Qualitative research has found an association between online portal use with increased patient satisfaction, engagement in their care, and perceived efficiency of in‐person visits.^4^, ^5^ Portal use can increase logistical efficiency and cost‐saving for both patients and providers.^6^, ^7^ Objective evidence on health outcomes with portal use has been mixed; there has been some evidence of improved disease‐related outcomes such as blood pressure and glycemic control and fewer emergency room visits and preventable hospital admissions, but it remains unclear to what extent these are confounded by demographic variation in portal use.^8^, ^9^, ^10^

Research across various specialties has suggested that the increase in patient portal use in recent years has not been uniform across all demographic groups. Multiple studies have found that male patients and older patients are less likely to use patient portals.^11^, ^12^ Racial disparities in patient portal use have been demonstrated, as have differences based on income and level of education.^13^, ^14^, ^15^, ^16^ However, to our knowledge these factors have not been studied in otolaryngology, and particularly not since the rapid expansion in interest in and use of online portals in the last several years. In this study, we aim to measure the frequency of use of an online patient portal for an otolaryngology practice at a tertiary care medical center and to analyze factors associated with varying levels of portal engagement.

## Methods

### Sample

This retrospective review queried all scheduled outpatient visits for Loyola University Medical Center's Department of Otolaryngology from December 2018 to December 2022. This project was fully approved by the Loyola University Medical Center Institutional Review Board (IRB#216604). Data were collected for scheduled clinic visits at 2 hospital‐based and 4 satellite ambulatory clinics. Clinic physicians included 3 general otolaryngologists, 5 head and neck oncology surgeons, 3 pediatric otolaryngologists, 1 laryngologist, 3 rhinologists, 5 otologists, and 1 facial plastic and reconstructive surgeon. Appointments with a midlevel provider were excluded, as were appointments with audiology or allergy only. New patient visits, return visits, and hospital follow‐up visits—all either in person or virtual—were able to be scheduled via portal. In‐office procedures and imaging studies were not able to be scheduled virtually. We defined the start of the COVID‐19 pandemic as March 2020, and all visits scheduled prior to March 2020 were classified as “pre‐COVID,” and those after March 2020 were classified as “post‐COVID.” We used the method of appointment scheduling as a proxy for measuring patient portal use. Data queried included appointment scheduling method (either in‐person or over the phone vs online self‐scheduling), type of clinic, and patient demographics.

### Statistical Analysis

*χ* ^2^ tests were used for univariate analyses. A binary logistic regression model was utilized for multivariate analysis. Predictors in the model consisted of demographics including (1) age in categories of <18, 18 to 65, and >65 years, (2) sex, (3) primary language spoken, (4) insurance payor type, (5) Loyola versus non‐Loyola primary care provider, and (6) appointment made pre‐ or post‐COVID. Binary variables were created for sex (male versus female), pre‐ versus post‐COVID, and institution of primary care provider (Loyola versus non‐Loyola). Odds ratios (ORs) and 95% confidence intervals were calculated for each covariate. All analyses were 2‐sided at the 0.05 significance level, and analyses were completed in SPSS Statistics 25 (IBM Corp Version 21.0).

## Results

From December 2018 to December 2022, there were 221,611 total clinic visits, with 49,462 unique patients. Of the unique patients, 15.9% scheduled at least 1 appointment using the online patient portal, and 7.5% of the total visits were scheduled using the portal. Portal usage significantly increased after the onset of the COVID‐19 pandemic. Prior to the onset of the pandemic, only 3.5% of unique patients had scheduled an appointment via the portal, and only 1.9% of the total pre‐COVID appointments were scheduled online. In comparison, 10.7% of post‐COVID appointments were scheduled using the portal (OR: 0.166, 95%, *P* < .01).

In univariate analysis, male patients were less likely than female patients to schedule appointments via the patient portal (*χ* ^2^ = 97.9, *P* < .01). Patients aged 18 to 64 were more likely to engage in portal use than those who were either under 18 or over 64 (*χ* ^2^ = 537.7, *P* < .0001). Use was lower in patients with primary language other than English (*χ* ^2^ = 250.0, *P* < .01) as well as patients with primary care physicians (PCPs) outside of the Loyola system (*χ* ^2^ = 1752.2, *P* < .01). There was not a statistically significant difference in portal scheduling across racial groups (*χ* ^2^ = 11.0, *P* = .051). In univariate analysis, patients with commercial insurance used the portal more than those with Medicare or Medicaid (*χ* ^2^ = 430.5, *P* < .01).

In multivariate analysis, female sex, age 18 to 65, English as the primary language, PCP within the Loyola system, and appointments made post‐COVID all remained significant predictors of the likelihood of patient portal use (Table 1). However, when controlling for those factors, patients with Medicare were more likely than those with commercial insurance to use the patient portal for scheduling (OR: 3.83, *P* = .03).

**Table 1:** Multivariate Analysis

Covariate	Odds ratio	95% CI	*P* value
Pre‐COVID	0.17	0.157‐0.176	<.01
Male	0.80	0.77‐0.83	<.01
Age 18‐65	‐	‐	‐
Over 65	0.59	0.55‐0.63	<.01
Under 18	0.89	0.82‐0.97	<.01
Outside PCP	0.46	0.44‐0.48	<.01
Non‐English language	0.53	0.49‐0.57	<.01
Commercial insurance	‐	‐	‐
Medicare	3.83	1.10‐13.30	.03
Medicaid	2.98	0.86‐10.35	.09

The odds ratio for the likelihood of appointment scheduling via the patient portal, with 95% CIs and *P* value.

Abbreviations: CI, confidence interval; PCP, primary care physician.

## Discussion

In this study, we show that online patient portal use in a tertiary care otolaryngology practice increased significantly after the onset of the COVID‐19 pandemic. In addition to the pandemic, which rapidly increased interest in and utilization of options for remote patient‐provider communication,^1^, ^2^, ^17^ this increase may have also been driven in part by the 21st Century Cures Act. This “information sharing rule” mandated that patients have access to medical records and test results in electronic formats at no charge to the patient and without significant delays. In response, organizations introduced technologies to make this available to patients, which in turn likely contributed to increased patient portal use among patients.

Our data show that patient portal use, while greatly expanded in the last 4 years, has not been equal among patients across demographic groups. Males, patients younger than 18 and older than 64, those with PCPs outside of the same medical system, and those with primary language other than English were all less likely to use the patient portal for appointment scheduling in multivariate analyses. Lower portal use in men, older patients, and those with a primary language other than English is consistent with findings from prior work in specialties outside of otolaryngology.^11^, ^12^, ^18^, ^19^, ^20^, ^21^ Our finding that portal use was less likely for patients younger than 18 is consistent with findings in primary care fields that patient portal adoption has been less robust for pediatric patients.^22^, ^23^ This is likely related in part to the increased logistical challenge of establishing portal access, as proxy access must be established by an adult guardian.

Prior work on disparities in patient portal use has generally demonstrated higher patient portal use among commercially insured patients.^24^, ^25^, ^26^ In univariate analyses, we find a higher percentage of remotely scheduled appointments among commercially insured patients; however, in our multivariate analyses, patients with Medicare had higher odds of portal use than commercially insured patients when controlling for other demographic variables. This may be explained by the fact that in this study we do not control for the level of medical comorbidity. Patients with Medicare may have more comorbid conditions,^27^, ^28^ and patients with more medical comorbidity and more interactions with the health care system have been shown in other specialty areas to be more frequent users of patient portals.^29^, ^30^, ^31^ Thus they may be more likely to have already established patient portal use by the time they present to our clinics.

While our study demonstrates that these disparities exist, it is also important to understand the underlying reasons for them. Technical ability to use an online portal is one consideration. For example, a patient portal that has its dashboard only in English may be difficult for patients with different primary languages to navigate.^20^ Patients may have structural barriers to access, such as lack of access to a computer or to internet service, or barriers such as visual or physical impairments which may make portal use difficult. Patients with less familiarity with technology and internet use may have a harder time setting up accounts and learning how to use a patient portal, which can contribute to the age disparity that we noted with portal utilization.^11^ However, it is important to recognize that lack of patient portal utilization may not only occur in patients who have barriers to access; some patients may not be interested in patient portal use either due to preference for in‐person communication or privacy concerns.^32^ A study of communication preferences for biopsy results in an otolaryngology clinic found that while most patients preferred remote means for notification, patients with lower health literacy had a stronger preference for in‐person discussion.^33^ Older patients in particular may also have more concerns about privacy when considering patient portal use.^32^ Furthermore, disparities may be driven by provider biases; Richwine et al showed that patient portal options were less likely to be discussed with black and Hispanic patients than white patients which lead to lower rates of use.^34^

In this study, we are limited by single‐center data; despite being obtained from a diverse, high‐volume otolaryngology practice, our results may not be generalizable to centers with different patient populations. In addition, we use appointment scheduling as a proxy for overall patient portal engagement. Patient‐specific data were available for appointment scheduling but not for other portal activities such as viewing results and private messaging with providers. While at the time of the study, approximately 75% of patients in our practice had registered for a patient portal account, significantly fewer use the portal for appointment scheduling. While the demographic patterns that we see in portal use for appointment scheduling is likely representative of patterns in other types of portal engagement, further work would be needed to determine whether these disparities persist in those areas as well.

Demonstration of these disparities in patient portal use provides an opportunity to guide improvements in patient outreach regarding options for remote health care engagement. Encouraging patients to sign up and utilize patient portals in the ambulatory clinic setting is important for improving patient engagement and health care outcomes. Patients are more likely to sign up and utilize patient portals when they are encouraged during their clinical encounters. Providers should also be cognizant of implicit biases and assumptions that may impact which patients they are more likely to discuss portal options with, as this can exacerbate disparities.^34^ Several effective strategies to promote patient portal adoption include communicating the advantages of using patient portals, training clinical staff to discuss the patient portal during the visit, simplifying the sign‐up process, on‐site assistance, as well as email reminders, and mobile options. At our institution, the patient portal sign up is embedded in the appointment check‐in process where a portal activation code is sent to the patient via text, email, or print form, and we have trained the clinical staff on strategies for how to encourage and help patients to activate the patient portal. The patient portal process has been simplified over the years and currently can be done instantaneously while the patient is in the clinic, or via email or text message activation link. In addition, we provide regular updates for physicians and advanced practice providers regarding patient portal engagement trends and portal sign up or interface updates.

On a broader level, these findings demonstrate the need for structural considerations on how to make patient portals more accessible. Effective and accessible communication between patients and providers is crucial for delivering high‐quality, patient‐centered care. Burks et al demonstrated significant gaps in the readability of patient education materials and health literacy levels of patients with head and neck cancer and pointed out the clear inequity this disparity creates.^35^ Similarly, we believe that health inequities are likely to be exacerbated if patient portals, which provide patients with multiple methods for being more engaged in their health care, are less accessible for certain populations or patients. The Healthy People 2030 framework set for by the US Department of Health and Human Services^36^ emphasizes 5 key areas to address and support in order to achieve its vision. These initiatives highlight the importance of addressing health disparities, health equity, social determinants of health, and health literacy. Particularly with the advent of the 21st Century Cures Act, providers have a mandate to provide patients with remote access to their records and results. This is not upheld if portals are difficult to use for certain demographic groups, so it is crucial to consider whether these remote options can be equitably accessed by patients who are, for example, older, have limited English proficiency, have lower technological literacy, or need a guardian to establish proxy access. Portal interfaces could be made more intuitive or come with more clear instructions for registration and use. Adding functionality in multiple languages is crucial for increasing accessibility. There may also be opportunities for health care organizations to partner with community services, such as public libraries, to educate community members on patient portals, their uses and advantages, and means of accessing them.

In addition to community resources, there have also been significant advancements in virtual tools which are starting to be incorporated in the outpatient setting. Clinic avatars, chatbots, and virtual assistants have the potential to be effectively utilized to increase patient portal engagement among patients in the ambulatory setting. These emerging digital tools will have the capability to provide round‐the‐clock assistance in helping patients navigate the portal and answer simple common questions, as well as providing language support, interactive tutorials, guided sign‐up, and reminders and notifications. In addition, these virtual tools can reduce the burden on health care staff by automating routine tasks and providing constant support to patients.

Patient portal use in otolaryngology is rapidly expanding and proves logistical and financial benefits for both patients and providers, and it is crucial to understand patient factors that may lead to lower utilization so that barriers can be addressed to mitigate disparities and make the technology broadly accessible to a diverse patient population. Emerging virtual tools have the potential to significantly enhance patient portal engagement in the outpatient setting by providing personalized guidance, support, and interactive experiences that cater to diverse patient needs and technological comfort levels.

## Author Contributions

**Jesse K. Siegel**, project conception, data analysis, writing, and revision of the manuscript; **Chloe Verducci**, data analysis, writing, and revision of the manuscript; **Agnes Hurtuk**, project conception, writing, and revision of the manuscript.

## Disclosures

### Competing interests

None.

### Funding source

None.
